# The microbial profile of a tissue necrosis affecting the Atlantic invasive coral *Tubastraea tagusensis*

**DOI:** 10.1038/s41598-021-89296-z

**Published:** 2021-05-10

**Authors:** Aline Aparecida Zanotti, Gustavo Bueno Gregoracci, Marcelo Visentini Kitahara

**Affiliations:** 1 Programa de Pós-Graduação Em Sistemas Costeiros E Oceânicos, Federal University of Paraná (UFPR), Curitiba, Brazil; 2 Center for Marine Biology, University of São Paulo (USP), São Paulo, Brazil; 3 Institute of Marine Sciences, Federal University of São Paulo (UNIFESP), Santos, Brazil

**Keywords:** Microbial communities, Microbiome

## Abstract

The Southwestern Atlantic rocky reef ecosystems are undergoing significant changes due to sun-corals (*Tubastraea tagusensis* and *T. coccinea*) invasion. At Búzios Island, on the northern coast of São Paulo State, where the abundance of *T. tagusensis* is particularly high, some colonies are displaying tissue necrosis, a phenomenon never reported for this invasive nor any other azooxanthellate coral species. Using next-generation sequencing, we sought to understand the relationship between *T. tagusensis* tissue necrosis and its microbiota. Thus, through amplicon sequencing, we studied both healthy and diseased coral colonies. Results indicate a wide variety of bacteria associated with healthy colonies and an even higher diversity associated with those corals presenting tissue necrosis, which displayed nearly 25% more microorganisms. Also, as the microbial community associated with the seven healthy colonies did not alter composition significantly, it was possible to verify the microbial succession during different stages of tissue necrosis (i.e., initial, intermediate, and advanced). Comparing the microbiome from healthy corals to those in early tissue necrosis suggests 21 potential pathogens, which might act as the promoters of such disease.

## Introduction

Due to the ever-increasing anthropogenic challenges, coral environments are being impacted by pollution, overfishing^1^, bleaching^2–4^, and fluctuations and changes associated with the corals’ symbiont community^5–11^. Such fluctuations and changes are disrupting the long-established species-specific relationships between corals and microorganisms like *Bacteria*, *Archaea*, eukaryotes, and viruses, ultimately resulting in several diseases in their coral hosts^5,7,9,11–15^.

Our understanding of the factors that influence coral's health and, consequently, its symbiont community, has progressed mainly through studies that determined the microbiota associated with healthy^16–19^ and diseased corals^7,11,14,15^. In brief, these studies suggested that there are fundamental microorganisms to the host and that the balance of the microbial community is the result of long (co-evolutionary) and short-term (ecological) processes acting simultaneously. Consequently, biotic and abiotic conditions that trigger coral stress unbalance such relationship, which, in turn, may lead to several host diseases^20^. Nowadays, there are over 20 diseases known to affect scleractinian corals^21^, but only a few had its pathogens identified^20^. Other studies have described the changes in the coral microbiota, indicating that the rise in the abundance of microorganisms from the genus *Ruegeria* leads to several coral diseases^10,11,14,22–24^.

In Brazilian waters, analyses of the microbial community have been performed on the endemic corals *Mussismilia braziliensis*^7,25^ and *Mussismilia hispida*^26,27^, as well as in other more widely spread species, such as *Madracis decactis*^26,28^, *Siderastrea stellata*^29^, and also on the invasive corals *Tubastraea coccinea*^26^ and *T. tagusensis*^30^. Some of these studies focused on the microbiota associated with healthy corals, but the microbial community associated with bleached and/or diseased colonies of *M. braziliensis, S. stellata,* and *M. decactis* were also determined. Nonetheless, besides diseases^7^ and acute bleaching events^31^, the Brazilian coral- and rocky-reef environments are being severely impacted by the invasion of sun-corals, *T. tagusensis,* and *T. coccinea*^32–34^*.* Currently, *T. tagusensis* is widespread in Brazil, particularly in São Paulo, Rio de Janeiro, Espírito Santo, Bahia, and Ceará states^35–40^. At Búzios Island, São Paulo State, a place that was known to harbor mainly *M. decactis*, *M. hispida*, *Palythoa caribaeorum*, turf, and sponges^41^, several rocky shores are saturated (100%) with invasive corals. Although several biological and ecological aspects are known to be key to the invasion success of *T. tagusensis,* it seems like they may be related to its reproductive characteristics, such as high production of planula^42^; early reproductive age^43^; clonality^44,45^; regeneration capacity^46^; and quick incubation—all hallmarks of opportunistic species^47^.

As a result of asexual planulae production, Southwestern Atlantic invasive *T. tagusensis* displays a high clonal rate^44^, and therefore a low genetic diversity, a phenomenon previously observed in other invasive species populations^48^. Such a decrease in diversity is caused by the founding effect—few specimens colonizing a new environment, which can reduce the adaptive potential of the species over time^48^. This condition of *T. tagusensis* may be the downside of this invasive species because, in addition to having a high rate of clonality, it also showed the absence of significant differences in the microbial community along a depth gradient^30^.

In 2014, when several rocky shores of the Búzios Island were already saturated with invasive corals, colonies of *T. tagusensis* displaying tissue necrosis were observed. Initially, affected colonies were seen in only one small location, but since then, affected colonies have become widespread. Such tissue necrosis is the first report of disease in azooxanthellate scleractinian corals. To better understand this tissue necrosis, here we characterize and compared its bacterial composition during different necrosis stages. Thus, the *T. tagusensis* microbiome was quali-quantitatively determined during the tissue necrosis progression, but a better understanding of the cause of such lesions requires further studies.

## Materials and methods

**Figure 1:**
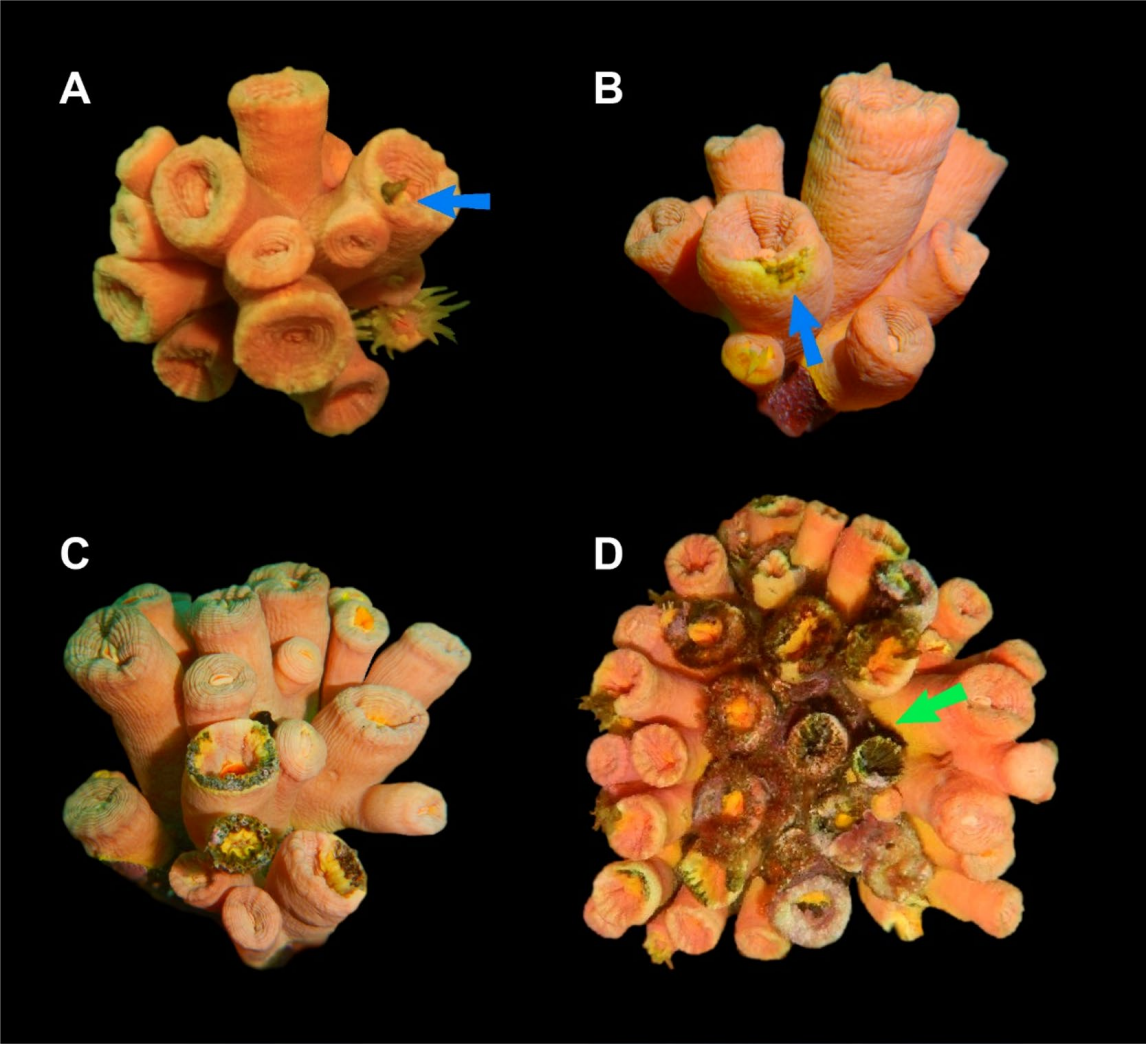
*Tubastraea tagusensis* colonies from Búzios Island—SP, showing signs of tissue necrosis. (**A**) and (**B**)—the early stage of tissue necrosis, with a single polyp displaying only a small brown dot (blue arrows). (**C**)—tissue necrosis progressing throughout the polyp (intermediate stage). (**D**)—Tissue necrosis at an advanced stage with polyps taken over by the disease (green arrow).

Eighteen specimens of the invasive coral *Tubastraea tagusensis* were collected in Búzios Island, on the northern coast of the São Paulo State, between 5 and 7 m deep. Sampling was carried out between August 28th and October 09th, 2017. All samples were readily frozen after collection. Part of the samples represented healthy coral colonies (n = 7), and the remaining specimens were from colonies displaying different stages of tissue necrosis: initial (n = 6) defined as colonies with a small necrotic region at the calicular margin (Fig. 1A, B); intermediate (n = 2) defined as polyps with more than 50% of the calicular margin affected (Fig. 1C); and advanced (n = 3). defined as fully necrotic polyps (Fig. 1D).

In all analyzed colonies (healthy and diseased) a fragment of approximately 25 mg (~ 5mm^2^) of the calicular margin containing tissue and skeleton was used for total genomic DNA extraction, for diseased colonies, the extracted portion was exactly the diseased area. DNA was extracted using the DNeasy Blood & Tissue kit (QIAGEN), following the manufacturer's instructions. The quality and purity of extracted DNA were analyzed through agarose gel electrophoresis (1,5%) and spectrophotometry (NanoDrop), respectively.

Using the universal bacterial primers 27F and 519R (V1-V3 region—LANE 1991; Turner et al. 1999), a fragment of the 16S rDNA with approximately 600 bp was amplified using the Advantage HF 2 PCR kit, following concentrations recommended by the manufacturer and two-stage cycling for diseased colonies, as follows: 94 °C for 1 min followed by 38 cycles at 94 °C for 30 s and 68 °C for 1 min. For healthy colonies, PCR reaction was conducted with the following cycling: initial denaturation step 94 °C for 60 s, followed by 30 cycles at 94 °C for 30 s, 56 °C for 40 s, and 68 °C for 33 s, with a final extension step at 68 °C for 33 s.

Amplicons were purified with magnetic beads (Agencourt AMPure XP) following the manufacturer's instructions and eluted in 50 µl of 1X TE buffer (10 mM Tris–HCl, pH 8, 1 mM EDTA). Final sample concentrations were measured using the Qubit dsDNA BR Assay Kit. Libraries were assembled with the NEBNext Ultra II FS DNA Library Prep Kit and their respective concentrations and size distributions were verified using the Qubit dsDNA HS (High Sensitivity) Assay Kit and the Bioanalyzer High Sensitivity DNA Chip, respectively. Sequencing was performed on the MiSeq platform (Illumina) using the MiSeq Nano v2 kit (500 cycles), at the Facility Center for Research from the University of São Paulo (CEFAP-USP). Sequences were deposited in the SRA database (PRJNA637639 and PRJNA675612).

Low-quality strands of each sequence, as well as short reads (< 50pb), were removed using SolexaQA++ software^49^. Identical sequences were grouped using the Swarm software with d = 1^50^ and then classified on the Mothur platform with a bootstrap cutoff of 80^51^ using the database 16S Silva—v.132 (SDB)^52^. Statistical analyses were performed using the STAMP—Statistical Analysis of Metagenomic Profiles software^53^, after the removal of eukaryotic, chloroplast, mitochondria, and unknown sequences. Statistical tests were performed for multiple groups through variance analyses (ANOVA) and post-hoc Turkey-Kramer tests and Benjamini–Hochberg FDR correction of multiple tests, being 0.01 the adopted p-value filter to identify the relative frequency of OTUs for each necrosis stage and their and their differences. The Whites' two-sided nonparametric t-test, CI method (DP bootstrap, Benjamini–Hochberg FDR multiple test correction, and *p*-value filter of 0.01) was applied to compare the microbiota between healthy colonies and those at the initial necrosis stage. The diversity parameters were derived from the classification tables (phylum, class, order, family, and genera), such as the wealth estimators Shannon, Neff Shannon, Simpson, and Neff Simpson (Simpson's inverse index). All *p*-values taken into account were the adjusted p-value after correction.

## Results

*Tubastraea tagusensis* tissue necrosis generally starts at the tissue from the calicular margin like a small brown dot (Fig. 1A, B) that later expands through the polyp (Fig. 1C), sometimes leading to the death of several polyps (Fig. 1D). When colonies with tissue necrosis were manipulated, the skeleton of the diseased region was significantly fragilized and brittle. From the tissue and skeleton of healthy and diseased corals, we obtained 738,462 classified sequences (averaging 220 bp) representing bacteria, chloroplasts, mitochondria, unknown, and eukaryotes. On average, the number of obtained taxonomic classifications from diseased corals (58,564) was four times higher than that from healthy corals (13,465). Sequences from eukaryotes, chloroplasts, mitochondria, and unclassified were removed from statistical analyses. The number of bacterial reads associated with target samples was, on average, 19,268 per coral colony displaying tissue necrosis, and ~ 1538 per healthy coral colony (Table 1).

**Table 1:** The number of classified sequences obtained from each analyzed stage of tissue necrosis.

Status	Sample		Total reads	RI	SHD	Neff Shannon/exp H	D	Neff/D2/Inverse Simpson
H	A		3,635	70	0.98	2.67	6.43E−01	1.55
H	B		1,939	70	1.07	3.05	6.04E−01	1.65
H	C		1,734	71	1.44	4.21	3.88E−01	2.58
H	D		1,110	113	2.73	15.27	1.77E−01	5.65
H	E		865	93	2.31	10.03	2.48E−01	4.04
H	F		793	73	1.89	6.63	4.10E−01	2.44
H	G		676	78	2.69	14.69	1.55E−01	6.46
DI	H		33,752	261	3.39	29.55	6.15E−02	16.26
DI	I		46,855	537	3.63	37.68	7.03E−02	14.22
DI	J		19,994	389	3.65	38.32	6.47E−02	15.45
DI	K		21,844	356	3.46	31.73	7.64E−02	13.09
DI	L		20,039	386	3.62	37.24	7.41E−02	13.49
DI	M		44,301	479	3.55	34.89	7.31E−02	13.68
DM	N		10,705	371	3.53	34.29	6.51E−02	15.36
DM	O		48,436	363	3.46	31.90	6.89E−02	14.50
DA	P		38,101	452	3.30	27.19	9.66E−02	10.35
DA	Q		24,489	514	3.82	45.74	6.16E−02	16.23
DA	R		28,106	325	3.35	28.55	8.31E−02	12.03

Status: Healthy (H); initial disease (DI); intermediary disease (DM); advanced disease (DA). Richness indices (RI), Shannon H diversity (SHD), Effective number of species (Neff Shannon/exp. H), Simpson index (D), Effective number of species (Neff/D2/inverse Simpson) based on the total reads found in each sample.

The richness index as well as the diversity and the effective number of species in both Shannon and Simpson indexes are higher in those colonies presenting tissue necrosis than that from healthy ones. Richness varies considerably between necrotic colonies (261–537), although there was no observable pattern between different necrosis stages. The greatest richness was found in advanced (514—sample Q) and initial (537—the sample I) stages of the disease. Regarding diversity, there is, on average, a fourfold increase in the number of effective genera from healthy to diseased colonies (Shannon Neff from 8.07 to 34.28 and Simpson Neff Simpson from 3.48 to 14.06).

**Figure 2:**
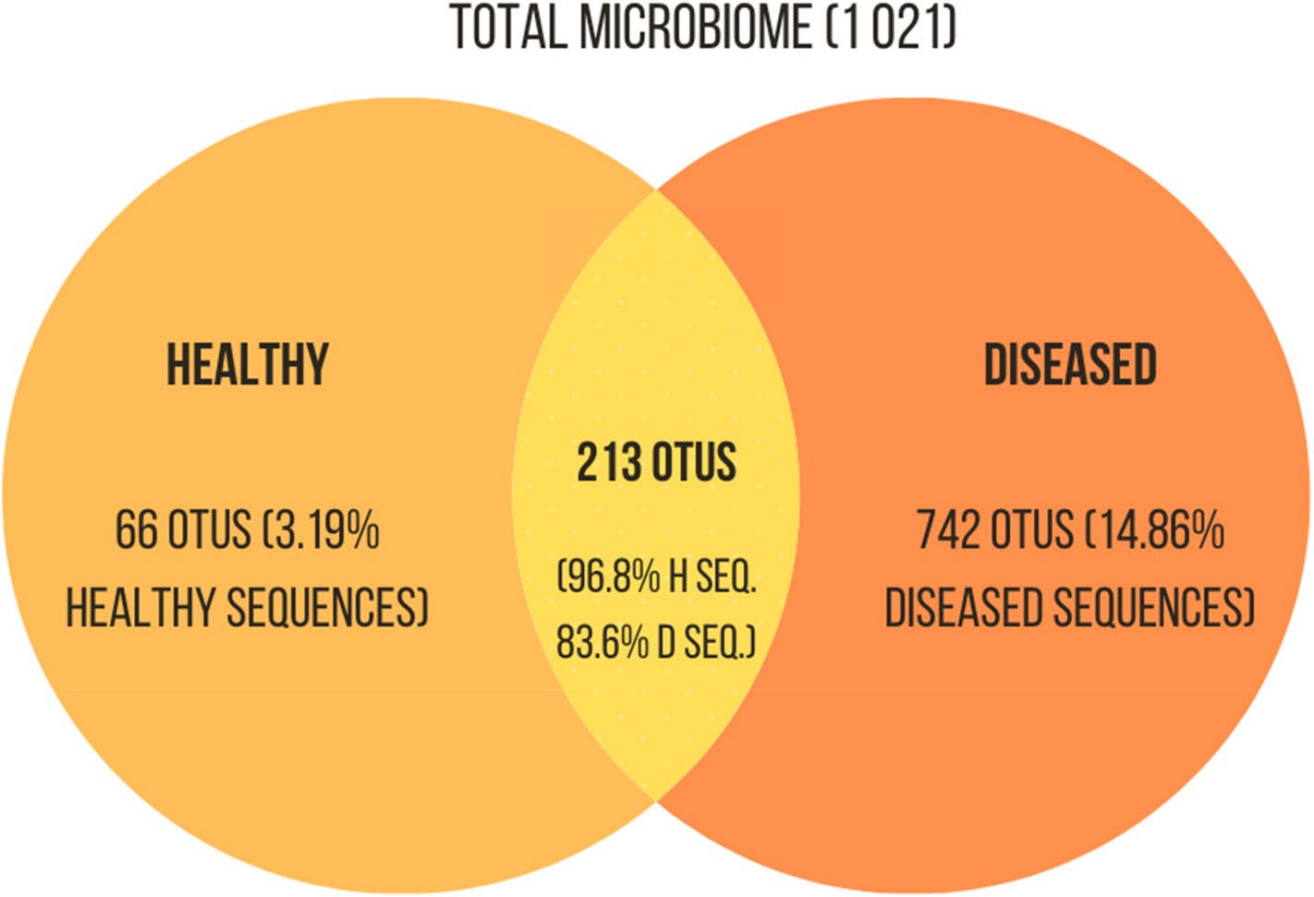
Venn Diagram indicating the number of OTUs of *Bacteria* associated with *Tubastraea tagusensis*. The microbiota exclusive to healthy colonies is represented in light orange, whereas the microbiota of diseased colonies is represented in dark orange. The intersection, yellow, indicates the microbiota shared by both groups.

Among *Bacteria*, 1,021 OTUs were detected, of which 213 occurred in both healthy and diseased colonies. These 213 common OTUs represent 96.8% of the microbiota abundance associated with healthy corals and an average of 83.6% of the abundance of microorganisms associated with diseased colonies. From the 1,021 identified OTUs, only 187 were more abundant in healthy corals, whereas the remaining predominated in diseased ones. In total, 742 genera were exclusively associated with diseased corals, representing 14.86% of their OTUs abundance, while 66 genera were exclusive to healthy colonies and represent 3.19% of their bacterial abundance (Fig. 2). Among the shared OTUs between healthy and diseased coral colonies, 47 displayed significantly different relative frequencies (< 0,01), and, of these, 25 were more frequent in healthy corals.

**Figure 3:**
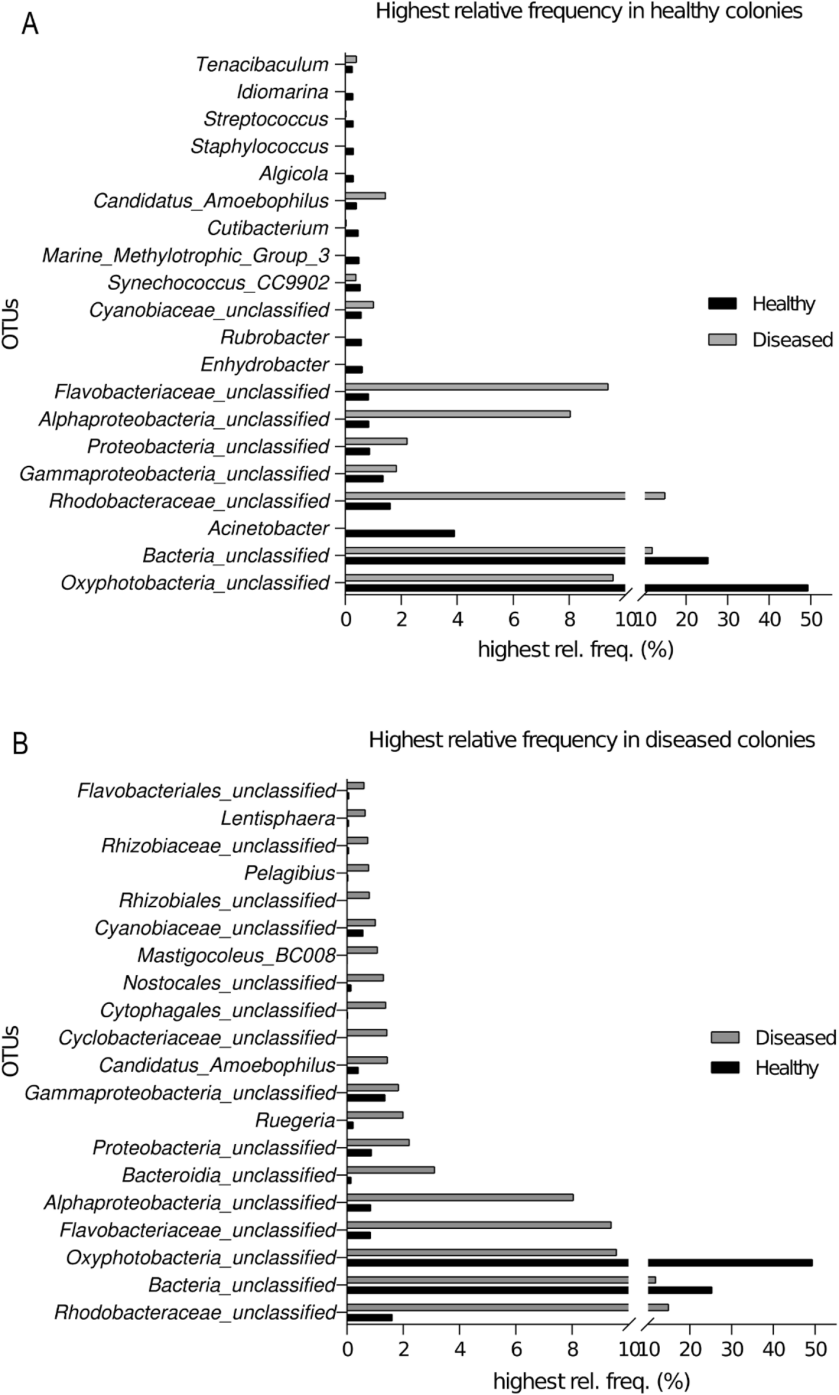
The 20 OTUs with the highest relative frequency in (**A**) healthy corals and their frequencies in diseased corals; and (**B**) associated with diseased corals and their frequencies in healthy corals. The figure was created using GraphPad Prism 8.0.2 (https://www.graphpad.com/).

At the genus level, even though most microorganisms have a relatively low frequency, it was possible to infer that some constitute most of the microbiome and markedly differ between coral conditions. In healthy colonies, 20 genera (Fig. 3A) represent 89.2% of the microbiota, with *Rubrobacter, Marine_Methylotrophic*, and *Idiomarina* being exclusive to them. The remaining OTUs are significantly abundant in diseased corals, although in a lower proportion (61.1%) of its associated community. Of the 20 most abundant OTUs in diseased colonies (Fig. 3B) (which encompass 74.1% of the detected microbiota), a single one was exclusive to diseased states (*Cyclobacteriaceae*_unclassified), while the remaining represent 82% of the microbiome associated with healthy colonies.

Comparisons of the microbiome from healthy and the observed stages of coral tissue necrosis suggest that 15 phyla compose nearly all the microbiota associated with *T. tagusensis.* Those same 15 phyla represent the majority of taxa, regardless of the disease condition and despite the presence of additional 22 phyla in the diseased colonies. On the other hand, the most abundant ones, *Cyanobacteria*, *Proteobacteria*, *Bacteroidetes*, *Actinobacteria*, and *Firmicutes* showed altered frequencies when compared between healthy and diseased colonies (e.g. increased frequency of *Proteobacteria* and *Bacteroidetes* and decreased frequency of *Cyanobacteria*—Table 2).

**Table 2:** The relative frequency of the major phyla of Bacteria (calculated with ANOVA) associated with healthy corals (H) and initial (DI), intermediate (DM), and advanced (DA) stages of tissue necrosis, and the *p*-value.

Phylum	H (%)	DI (%)	DM (%)	DA (%)	*p*-value
*Cyanobacteria*	50.54	15.81	20.45	20.4	5.11E−02
*Proteobacteria*	17.87	45.83	32.78	35.92	2.15E−01
*Bacteroidetes*	2.81	22.37	29.66	24.98	6.53E−03
*Actinobacteria*	1.82	0.83	0.31	1.38	9.45E−04
*Firmicutes*	0.99	0.11	0.01	0.04	4.29E−01
*Planctomycetes*	0.6	2.33	2.04	2.02	1.62E−01
*Verrucomicrobia*	0.22	0.18	0.26	0.37	4.67E−02
*Epsilonbacteraeota*	0.14	0.002	0.02	0.02	7.70E−01
*Lentisphaerae*	0.09	0.62	1.46	0.91	5.82E−01
*Chloroflexi*	0.08	0.34	0.47	0.32	2.17E−01
*Fusobacteria*	0.07	0.03	0.16	0.09	2.15E−01
*Nitrospinae*	0.04	0.01	0.03	0.04	4.41E−01
*Acidobacteria*	0.03	0.28	0.08	0.24	7.84E−01
*Nitrospirae*	0.02	0.02	0.03	0.05	2.14E−01
*Kiritimatiellaeota*	0.003	0.04	0.07	0.05	7.47E−01
Unclassified Bacteria	24.7	10.77	11.89	12.84	2.75E−01
Total	100	99.57	99.69	99.68	

**Figure 4:**
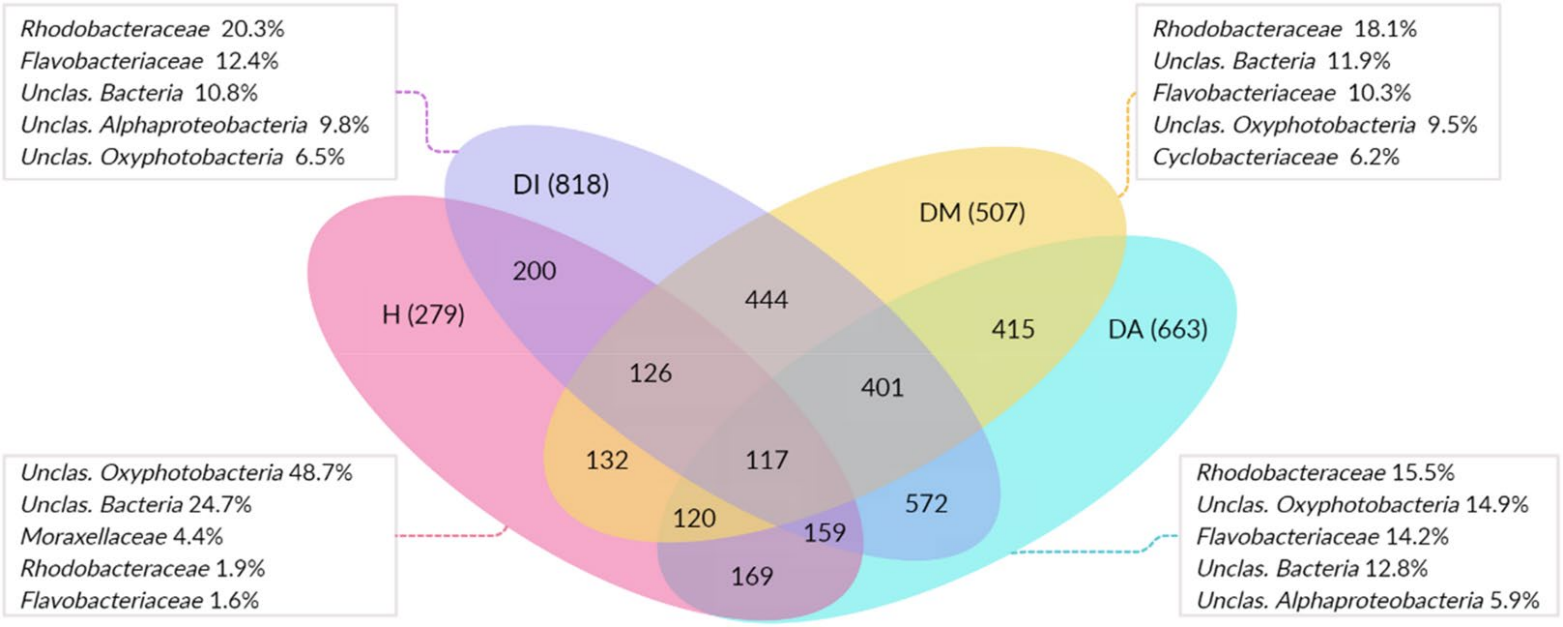
Venn diagram showing the microbiota (OTUs) of each analyzed stage, of which pink represents healthy colonies (H), purple initial necrosis stage (DI), yellow for colonies in the intermediate necrosis stage (DM), and blue represent colonies in the advanced stage (DA). The numbers of shared OTUs between stages are shown in the intersections. Within the boxes are the five most abundant classifications at the family level for each corresponding group.

When monitoring the microbial succession in the analyzed stages of tissue necrosis, it is clear that most Bacterial groups present in the microbiome of healthy colonies are also in diseased ones. When the initial signs of tissue necrosis are observed (initial phase), the richness and total reads have a significant increase, but 85.4% (200 OTUs) of microorganisms identified in healthy colonies are also identified in this stage. In the intermediate stage of infection, the number of OTUs increases if compared to healthy colonies (Fig. 4) but decreases 39% concerning the initial stage of the disease. In advanced necrosis stages, a rise in microorganism abundance is observed when compared to the intermediate stage, but 85.4% of the microbiota (169 OTUs) are also found in healthy colonies. When comparing tissue necrosis stages, there is a higher OTUs richness shared between all stages of the disease (Fig. 4), of which 284 OTUs are absent in healthy coral colonies. This group of OTUs represents 12.63%, 18.98%, and 13.41% of abundance from the initial, intermediate, and advanced necrosis stages respectively.

Despite the shared microorganisms for each stage, including healthy colonies, a group of bacteria exclusive to each sample is observed: 66 in healthy colonies; 171 in colonies in initial necrosis stage; 43 for the intermediate stage; and 67 in colonies with advanced tissue necrosis.

The microbial core (persistent microorganisms found in all specimens of a particular holobiont species) of *T. tagusensis* is comprised of 8 genera (Zanotti et. al 2020). Of these, *Rubrobacter* is absent in all necrotic colonies, *Acinetobacter* is absent in the intermediate stage, *Enhydrobacter* is absent in the intermediate and initial stages, and *Hydrogenophilus* is absent in intermediate and advanced tissue necrosis stages. The microbial core is responsible for only a part of the microbial community of healthy colonies (6.68%), but it is significantly lower in comparison to that from diseased colonies, representing only 2.98%, 2.22%, and 1.39% in colonies at initial, intermediate, and advanced stages respectively. *Ruegeria* was more frequent in diseased colonies, representing 2.5%, 1.45%, and 1.15% of the microbiota associated with the tissue necrosis initial, intermediate, and advanced stages, respectively (Table 3).

**Table 3:** Mean relative frequencies of genera that comprise the microbial core in healthy and at the initial (DI), intermediate (DM), and advanced (DA) stages of the disease in *Tubastraea tagusensis*.

Genera	Relative frequencies (%)			
	Healthy	DI	DM	DA
*Ruegeria*	0.22	2.55	1.45	1.15
*Cutibacterium*	0.46	0.07	0.01	0.02
*Rubrobacter*	0.57	0	0	0
*Acinetobacter*	3.78	0.004	0	0.001
*Enhydrobacter*	0.59	0	0	0.001
*Hydrogenophilus*	0.22	0.008	0	0
*Staphylococcus*	0.29	0.005	0.002	0.003
*Synechococcus_CC9902*	0.52	0.34	0.76	0.21
Total	6.65	2.97	2.22	1.38

To identify potential bacterial triggers of tissue necrosis in *T. tagusensis*, special attention was given to the community of microorganisms associated exclusively with the initial stage of the disease, and its comparison to those from healthy coral colonies. Among them, 65 significant differences were observed (*p* value between 0 and 0.0096), but genera that were not linked to disease were excluded, such as those more frequent in healthy colonies (4) and those that were not detected in all diseased colonies in the initial stage (30). Therefore, 31 OTUs fitted these criteria, of which only 13 were classified to the genus level. To improve robustness, genera with less than 10 reads per colony were excluded from further characterization as disease potential triggers. Finally, besides being identified in all colonies during the initial stage of necrosis, these 21 OTUs (Table 4) were also present in all subsequent stages of the disease.

**Table 4:** Classification of the 21 OTUs found in all colonies presenting necrosis and with a tendency of increasing relative frequency as the disease progresses in the coral *Tubastraea tagusensis*.

Family	Genus	Healthy	DI	*p* value
*Alphaproteobacteria_unclassified*		0.847	10.08	7.24E−04
*Amoebophilaceae*		0.052	0.62	2.92E−03
*Arenicellaceae*	*Arenicella*	0.013	0.524	1.81E−03
*Arenicellaceae*		0.004	0.112	1.21E−03
*Bacteroidia_unclassified*		0.15	2.633	5.79E−04
*Cyclobacteriaceae*		0	0.529	1.26E−03
*Deltaproteobacteria_unclassified*		0.004	0.183	2.66E−03
*Flavobacteriaceae*		0.841	9.367	6.21E−04
*Flavobacteriaceae*	*Spongiibacterium*	0	0.15	2.75E−03
*Flavobacteriales_unclassified*		0.07	0.53	1.75E−03
*Kiloniellaceae*		0	0.114	1.77E−03
*Myxococcales_unclassified*		0	0.124	4.11E−03
*Nostocales_unclassified*		0.154	1.488	6.86E−03
*OM190_fa*		0.017	0.219	2.02E−03
*Phormidesmiales_unclassified*		0	0.526	4.04E−03
*Proteobacteria_unclassified*		0.88	2.391	7.85E−03
*Rhizobiales_unclassified*		0.004	0.849	7.87E−03
*Rhodobacteraceae*		1.613	15.88	0
*Rhodobacteraceae*	*Ruegeria*	0.226	2.589	1.30E−03
*Rhodobacteraceae*	*Epibacterium*	0.004	0.727	6.72E−03
*Sphingomonadaceae*	*Sphingorhabdus*	0.023	0.298	7.67E−04

## Discussion

Changes in the microorganism community associated with the colonies of the invasive coral *Tubastraea tagusensis* are significant from the first signs of tissue necrosis. Such changes lead to increased microbial diversity, richness, and the effective number of genera (e.g. bacteria), which is compatible with previous studies on coral diseases^54–57^. However, most of the microbiota associated with diseased corals (~ 83.6%; 213 OTUs) is present in healthy colonies. Thus, despite the detection of nearly 750 OTUs exclusive in diseased colonies, they did not replace the microbiota associated with healthy hosts but instead reduced it as they grew. Despite the community shift, the relative frequency of bacterial OTUs found exclusively in diseased colonies is low, with the highest disease-specific genus composing 5.1% of the total microbiota in the intermediate stage (unclassified *Cyclobacteriaceae*). Such low frequency may indicate that the majority of the microbiota in diseased corals is composed of opportunistic species and/or secondary colonizers^55,56^, which survived in the coral due to the imbalance of the microbial community caused by the primary infection. However, the opposite has also been detected (e.g., 63 OTUs present only in healthy colonies, all with low relative frequencies [an average of ~ 0.097%, representing 3.19 of the total abundance]).

We also observed that the necrotic affected area is not directly related to the diversity of associated microorganisms. In the initial stage, in which it has less than 0.25 cm^2^, we identified a greater diversity of microorganisms. Also, this is a stage of tissue necrosis that has the most exclusive diversity, suggesting candidate triggers of the disease. In the intermediate stage, which displayed an enlargement of the area affected by the necrosis (Fig. 1C), the number of identified OTUs is lower compared to that from the onset of the disease, possibly indicating a holobiont response. At the advanced stage, the number of identified genera is higher than that in the previous stage, but such increment is not significant compared to the beginning of the infection. Such a variation in the microbiome indicates a rapid destabilization of the symbiont community during the initial stages of *T. tagusensis* infection, and subsequent colonization by opportunistic microorganisms, as observed in other diseases from zooxanthellate counterparts^55,58^.

In parallel to the microbiome related to necrosis, attention was given to the microbial core. Among the eight genera considered to be part of the microbial core of *T. tagusensis* (Zanotti et al. 2020), four were not found in colonies presenting tissue necrosis (Table 3). Such a difference might be a result of the microbiome imbalance caused by the disease. Nonetheless, the relative frequency of four microbial core genera was significantly lower in polyps presenting necrosis. However, *Ruegeria* had different patterns once it was the most abundant genera in diseased colonies. This genus is known to be associated with several healthy^14,59–61^ and diseased zooxanthellate corals^10,11,14,22–24^. Previous studies suggested that *Ruegeria* inhibits/controls the growth of other bacteria genera through tropodithietic acid^62^. Also, *Ruegeria* inhibited a widely known opportunistic coral pathogen, *Vibrio coralliilyticus*^63^.

Statistical analyses of the microbiome also indicate that within the bacterial OTUs identified, 21 (Table 4) might represent pathogenic ones. Of these, only five were classified to genus level, one of them being *Ruegeria*, which as mentioned has no pathogenic profile. Within the remainder, only *Arenicella* has been associated with coral disease (skeletal growth anomalies in *Platygyra carnosa*^23^), although the herein detected *Sphingomonadaceae* has also been described as a putative pathogen associated with *Acropora cervicornis* and *A. palmata* disease^64^. Besides that, although there is no previous report of *Epibacterium* in relationship to scleractinian corals (a group described associated with seaweed surfaces^65^), its detection and increased abundance during *T. tagusensis* tissue necrosis may be related to the exposure of the coral skeleton to the environment. As the necrosis advances, the exposed skeleton becomes a potential substrate for several organisms, like filamentous algae.

Apart from the aforementioned OTUs, several remained unclassified at the genus level. Among them, the *Flavobacteriaceae* and *Rhodobacteriaceae* were found in high abundance in *T. tagusensis* tissue necrosis and were previously associated with the following scleractinian diseases: White band^55^; White plague^7,66^; Yellow-band^14^; White syndrome^67^; White-spot syndrome in *Porites*^11^; White Plague^66^; stony coral tissue loss disease^68^; and Black band^10^. Additionally, the skeleton fragility in those colonies affected by the tissue necrosis may be related to a higher frequency of *Mastigocoleus*, a bacterial genus known to have bioerosion capabilities^69^.

Although concerning as another disease is reported to a scleractinian coral, the described tissue necrosis brings a glimpse of hope in the face of *T. tagusensis* unprecedented bioinvasion and spreading in the Southwestern Atlantic. Thus, despite its invasion capacity, the founding effect^44,70^, and the associated microbial community without major significant differences might be a disadvantage for this species, making it highly susceptible to diseases as the tissue necrosis reported herein.
